# For Melancholia

**Published:** 1886-02

**Authors:** 


**Melancholia**.—Dr. A. S. Defoe, of Washington, N. J., writes, that he has found the following to give excellent results in melancholia: R. Valerianates of zinc, quinine and iron each twenty grains, to be divided into twenty pills. One pill is to be taken three times a day before meals. The drugs should be absolutely pure. Dr. Defoe says he has tried this remedy thorougly, and finds it a specific for the worry of nervous women and for incipient melancholia.—*Med. Rec.*

